# An investigation of antioxidative and anti-inflammatory effects of *Taraxacum coreanum *(white dandelion) in lactating Holstein dairy cows

**DOI:** 10.5455/javar.2024.k781

**Published:** 2024-06-08

**Authors:** Sung Jae Kim, Young Jun Jo, Sang-Hee Jeong, Yo-Han Kim, Jeong Hee Han

**Affiliations:** 1 Department of Companion Animal Health, Kyungbok University, Namyangju, Korea; 2 College of Veterinary Medicine and Institute of Veterinary Science, Kangwon National University, Chuncheon, Korea; 3 Department of Biomedical Laboratory Science, College of Life and Health Science, Hoseo University, Asan, Korea

**Keywords:** Antioxidant, dairy cow, proinflammatory cytokine, somatic cell count, *Taraxacum coreanum*

## Abstract

**Objective:**

The aim of this investigation was to examine the impact of *Taraxacum coreanum* (known as dandelion) (TC) and TC mixtures with milk thistle (MT) or *Aspergillus oryzae* (AO) as feed additives on the immune response, milk quality, and milk production in Holstein cows over 6 weeks of administration.

**Materials and Methods:**

Thirty-two healthy Holstein dairy cows were provided 30 kg of total mixed ration (TMR) with no TC, 90 gm TC, 54 gm TC + 36 gm MT, or 54 gm TC + 36 gm AO 40% groups. The feed additives were supplied daily in two equal portions (per 45 gm) by topdressing the TMR for 6 weeks. Milk and blood samples were collected weekly.

**Results:**

In the TC-treated cows (TC, TC + MT, and TC + AO groups), significantly lower peripheral blood white blood cell (WBC) counts at 6 weeks and milk somatic cell counts (SCCs) at 4–6 weeks of administration were observed. Concentrations of serum superoxide dismutase (SOD) and glutathione peroxidase (GSH-px) were notably elevated in cows treated with TC for 4–6 weeks, while levels of proinflammatory cytokines concentrations of tumor necrosis factor-alpha (TNF-α) and chemokine (IL-8) were significantly reduced in TC-treated cows after 3–6 weeks of administration.

**Conclusion:**

These results suggested that TC or a TC mixture with other medicinal herbs supplementations enhanced the serum antioxidative activities and, consequently, might suppress the adverse immune response due to lower serum TNF-α and IL-8 release supported by lower WBC and SCC counts.

## Introduction

Recently, various substances, including antibiotics, hormones, mineral supplements, and immune enhancers, have been widely used to improve milk production in the dairy cow industry [1]. Although various substances have been used as growth promoters, their overuse may lead to undesirable side effects directly affecting human health [1]. Alternatively, medicinal herbs are considered promising options for improving the physiological performance of animals. Taraxacum, also known as dandelion, is a large genus of perennial flowering plants belonging to the *Asteraceae* family. In traditional medicine, they have been used for relief of inﬂammation and rheumatism [2], and neuroprotective, hepatoprotective, anti-cancer, antioxidant, anti-inflammatory, and anti-microbial activities were also revealed in humans [3–8]. Among the Taraxacum species, *Taraxacum coreanum *(TC), also called “white dandelion,” is a native domestic species in Eastern Asia, including South Korea, Japan, and China, and widespread in South Korea. TC is known to have various beneficial components such as polysaccharides, taraxasteryl acetate, daucosterol, b-sitosterol, chrysoeriol, diosmetion, esculetin, luteolin, luteolin-7-glucoside, 5-hydroxypyttolidin-2-one, taraxinic acid, and taraxinic acid-1-β-D-glucopyranoside [4,9]. Particularly, TC was found to contain a higher quantity of phenolic compounds and to exhibit greater antioxidant activity compared to *Taraxacum officinale*, commonly known as dandelion [8].

Milk thistle (MT) is a well-known herb that has been used in herbal preparations for centuries. Silymarin, a primary active constituent of MT, enhances antioxidant levels and improves the prognosis of hepatic ailments induced by oxidative damage [10]. It is employed in agricultural sectors as a dietary supplement to ameliorate growth performance, oxidative resilience, and liver functionality throughout the productive life span [11]. *Aspergillus oryzae* (AO) is utilized as a probiotic in livestock, serving as a significant source of various enzymes such as glucoamylase, alpha-amylases, and proteases, thereby potentially enhancing digestive efficacy when incorporated as a feed additive [12]. The polysaccharides present in the yeast cell wall not only interact with immune cells for immune enhancement but also have the ability to bind bacteria, thus averting attachment and colonization of pathogens in the gastrointestinal tract [13]. Therefore, we investigated the effect of TC and TC mixtures with MT or AO as feed additives on the immune response, milk quality, and milk production in Holstein cows over 6 weeks of administration.

## Materials and Methods

All experimental procedures involving rats were conducted following the approved protocols of the Hoseo University Laboratory Animal Care and Use Committee (HUACUC-18-166(1); Asan-si, South Korea). Similarly, all procedures involving cows were carried out in accordance with the protocols approved by the Kangwon National University Laboratory Animal Care and Use Committee (KW-230807-1; Chuncheon-si, South Korea).

### Preparation of substances for feed additives

Three types of feed additives with TC as a main ingredient and MT and AO as additional ingredients (TC, TC + MT, and TC + AO), were prepared. TC and MT were cultivated on a plant farm located in Kang-won Province, South Korea. The aerial parts (flowers, leaves, and stems) of TC and MT were harvested around early May, naturally dried, and powdered. To prepare AO, whole wheat flour inoculated with AO was fermented at 40°C for 10 days, whereafter the fermented products were dried, powdered and at stored at 4°C.

### Assessment of the oral ingestion acute toxicity in rats

Before TC administration to Holstein cows, acute ingestion toxicity was assessed in rats. All procedures were performed according to the protocols announced by the Animal and Plant Quarantine Agency, South Korea (Guidelines for Acute Toxicity Assessment of Animal Drugs; No. 2016–22). For the acute toxicity assessment, a mixture of 990 gm of TC and 10 gm of cyproheptadine (appetite stimulant) was prepared. Twenty rats (10 females and 10 males, 8 weeks of age) were divided into control (*n =* 10) and assessment (*n =* 10) groups with equal sex ratios. The maximum administration dose of 2,000 mg/kg/body weight was determined according to the Organization for Economic Cooperation and Development test guideline 401 acute oral toxicity (1987.02.24). The dose was determined based on the body weight of the rats on the day of administration. After 12 h of fasting, a single oral administration of the mixture was administered to the assessment group, and sterilized normal saline was administered to the control group. Feed was supplied again 4 h after administration. Before and 14 days after administration, clinical signs and death were confirmed, and an autopsy was performed to identify any histopathological changes.

### Experimental design

The experiment was conducted on a commercial dairy farm located in the Chung-Nam Province, South Korea. A total of 32 healthy multiparous Holstein dairy cows (45–50 months of age) in the late lactation stage were randomly selected and evenly assigned to four groups with access to water throughout the study period. The total mixed ration (TMR) was supplied twice daily (08:00 and 20:00). The feed additives were prepared as follows: TC alone (TC 100%), TC + MT (60% TC and 40% MT), and TC + AO (60% TC and 40% AO). The animals were fed 30 kg of TMR with no TC (G1 group; *n =* 8), 90 gm of TC (G2 group; *n =* 8), 54 gm of TC + 36 gm of MT (G3 group; *n =* 8), or 54 gm of TC + 36 gm of AO (G4 group; *n =* 8). The feed additives were supplied daily in two equal portions (45 gm per portion) by top dressing on the TMR. The composition of TMR is listed in Table 1.

### Quality analysis of milk

Milk was collected before and after the administration of feed additives once a week for 6 weeks to analyze the milk quality of each experimental group. Milk protein, milk fat, lactose, solids not fat (SNF), and somatic cell count (SCC) were analyzed.

### Complete blood cell count (CBC) and serum chemistry

Blood was collected from the jugular vein of the cows once a week for six weeks, i.e., before and during the administration of the feed additives, by drawing 1 ml of blood into a potassium EDTA tube. Hematological parameters including white blood cells (WBCs), red blood cells (RBCs), hemoglobin (HGB), hematocrit (HCT), mean corpuscular volume (MCV), mean corpuscular HGB (MCH), MCH concentration (MCHC), and platelet count (PLT) were assessed using a hematology analyzer (Urit–3,000 Vet plus Hematology Analyzer, Urit Medical Electronic).

**Table 1.** Nutrient composition of the TMR on a DM basis.

Items	TMR (DM basis %)
Moisture (%)	34.37
Crude protein (%)	16.55
Crude fat (%)	4.10
Crude ash (%)	9.01
Crude fiber (%)	15.52
Nitrogen-free extract (NFE, %)	54.82
Neutral detergent fiber (NDF, %)	42.79
Acid detergent fiber (ADF, %)	23.15
Total digested nutrient (TDN, %)	71.55
Metabolic energy (ME, Mcal/kg)	2.50
Net energy lactation (NEL, Mcal/kg)	1.64
Calcium (Ca, %)	0.81
Phosphorus (P, %)	0.48

The serum was separated from a coagulated blood sample for serum chemistry analysis. Biochemical parameters including alanine aminotransaminase (ALT), aspartate transaminase (AST), lactate dehydrogenase (LDH), alkaline phosphatase (ALP), total protein (TP), albumin (ALB), total bilirubin (T-BIL), glucose (GLU), total cholesterol (T-CHO), blood urea nitrogen (BUN), creatinine (CRE), and triglyceride (TG) were measured using a blood chemistry analyzer (7,020 Automatic Analyzer, HITACHI).

### Antioxidative activity and proinflammatory cytokine and chemokine analyses

Antioxidative activities of serum and milk were evaluated using the collected serum and milk samples. The concentrations of the antioxidant enzymes superoxide dismutase (SOD) and glutathione peroxidase (GSH-px) in serum samples were measured using commercial ELISA kits (Bioassay Technology Laboratory, UK) following the manufacturer’s protocol.

The status of the inflammatory response was evaluated by measuring the concentration of proinflammatory cytokines in serum samples. Concentrations of tumor necrosis factor-alpha (TNF-α) and interleukin-8 (IL-8) were measured using ELISA kits (Bioassay Technology Laboratory) following the manufacturer’s protocol.

### Statistical analysis

Statistical analyses were conducted using R Studio v4.3.0. (R Tools Technology, Canada). One-way ANOVA followed by the Tukey HSD multiple comparison method was utilized to assess differences in values among the groups. Statistical significance was determined at *p* < 0.05.

## Results

### Acute toxicity assessment of TC in rats

There were no abnormal body conditions or particular histopathological signs before or after 14 days of administration in any of the rats. Therefore, the lethal dose of TC was above the maximum administered dose, confirming the safety of TC.

### Milk yield and composition

**Figure 1.**
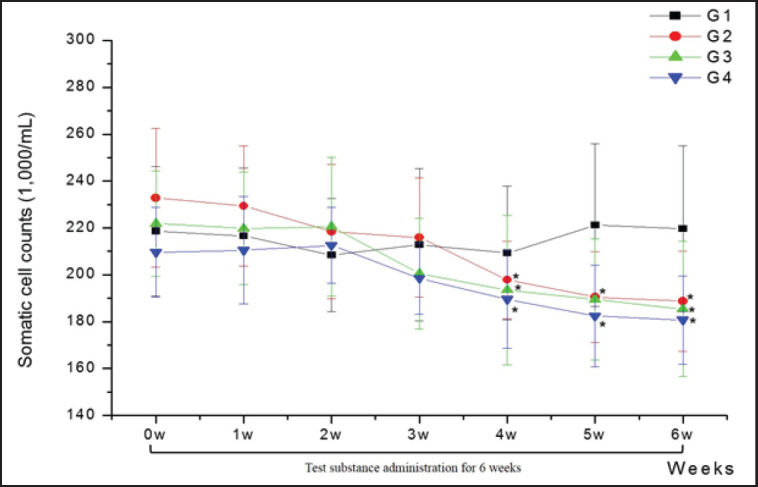
Changes in SCCs in Holstein cows with no TC (G1; *n =* 8), 90 gm TC (G2; *n =* 8), 56 gm TC + 36 gm MT (G3; *n =* 8), 56 gm TC + 36 gm AO (G4; *n =* 8) during the 6 week administration period.

Analysis of the effect of each feed additive on milk yield and quality revealed no significant differences in fat, protein, lactose, or non-fat solids in milk (Table 2). However, in the case of somatic cells in milk, the TC-treated groups (G2, G3, and G4) exhibited significantly lower SCC than the control group after 4 to 6 weeks of administration (*p* < 0.05; Fig. 1).

### CBC and serum chemistry

Clinical symptoms were not observed in the cows before, during, or after the experimental period. The results of CBC and serum chemistry analyses were within the reference range, and there was no significant difference among the groups during the 6-week administration period, except for the WBC count (Tables 3 and 4). In the WBC count, all groups were relatively high from the start compared to a previous report [14], and the G1 group maintained similar levels during the 6-week administration period. However, the TC-treated groups (G2, G3, and G4 groups) showed a continuous decreasing tendency until 5 weeks of administration (*p* > 0.1), and the WBC count was significantly (*p* < 0.05) lower in the TC-treated groups (G2, G3, and G4 groups) at 6 weeks of administration than in the control group (Table 3).

### Antioxidative activity and inflammatory response

**Figure 2.**
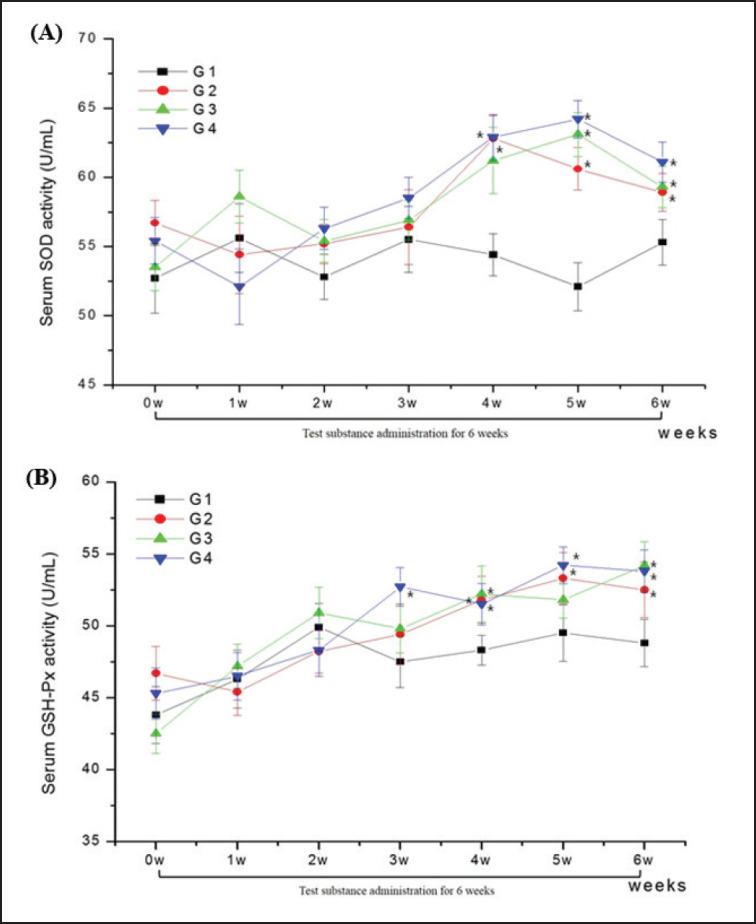
Changes in serum concentrations of SOD (A) and GSH-px (B) in Holstein cows with no TC (G1; *n =* 8), 90 gm TC (G2; *n =* 8), 56 gm TC + 36 gm MT (G3; *n =* 8), 56 gm TC + 36 gm AO (G4; *n =* 8) during the 6 week administration period.

In terms of serum antioxidative activities, the TC-treated groups (G2, G3, and G4) exhibited significantly (*p* < 0.05) higher SOD and GSH-px concentrations than the control group (Fig. 2). In the SOD, G2, and G4 groups after 4 weeks, and G2, G3, and G4 groups after 5 and 6 weeks of administration, the levels were significantly (*p* < 0.05) higher than those in the control groups (Fig. 2A). In the GSH-px group, the G4 group after 3 weeks, G2 and G4 after 5 weeks, and G2, G3, and G4 groups after 4 and 6 weeks of administration showed significantly (*p* < 0.05) higher concentrations compared with the control group (Fig. 2B).

**Figure 3.**
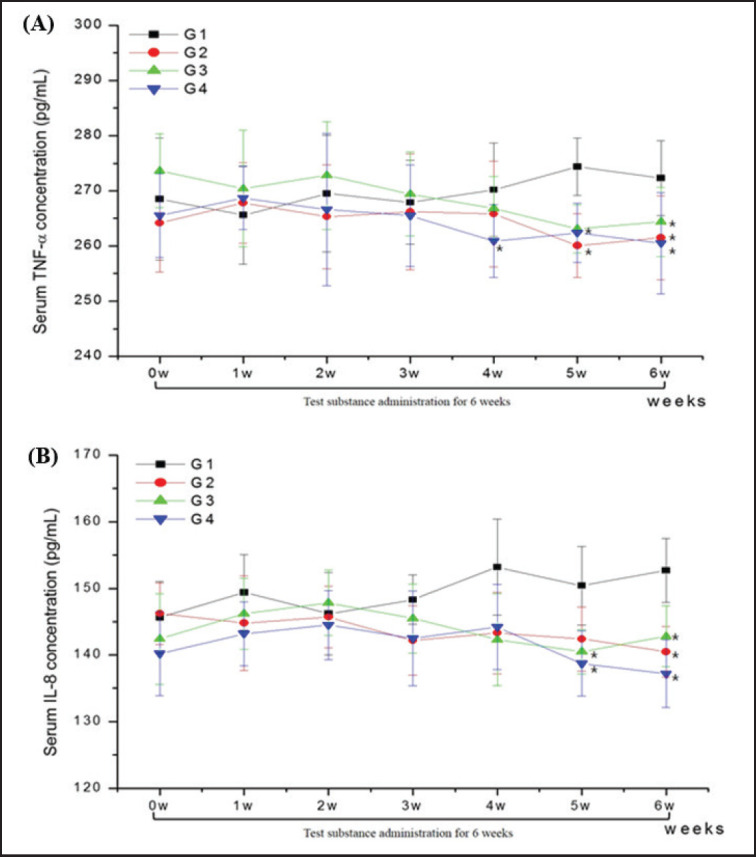
Changes in serum concentration of TNF-α (A) and IL-8 (B) concentrations in Holstein cows with no TC (G1; *n =* 8), 90 gm TC (G2; *n =* 8), 56 gm TC + 36g MT (G3; *n =* 8), 56 gm TC + 36 gm AO (G4; *n =* 8) during the 6 week administration period.

In the serum proinflammatory response, the TC-treated groups (G2, G3, and G4) exhibited significantly lower TNF-α and IL-8 concentrations than the control group (Fig. 3). In the TNF-α, the G4 group at 4 weeks, G4 and G2 groups at 5 weeks, and G2, G3, G4 groups after 6 weeks of administration showed significantly (*p* < 0.05) lower concentration compared with the control group (Fig. 3A). For IL-8, the G3 and G4 groups at 5 weeks and the G2, G3, and G4 groups after 6 weeks of administration showed significantly (*p* < 0.05) lower concentrations compared with the control group (Fig. 3B).

**Table 2.** Changes in milk yield and composition in Holstein cows during the 6-week administration period.

Parameters	Group*	0w	1w	2w	3w	4w	5w	6w
Milk yield (kg)	G1 (control)	21.24 ± 3.53	21.36 ± 2.74	21.84 ± 4.14	20.78 ± 3.45	21.54 ± 2.79	21.65 ± 3.86	21.12 ± 3.59
	G2 (TC)	20.18 ± 4.27	21.45 ± 2.42	21.63 ± 3.43	21.44 ± 4.25	22.08 ± 2.65	21.82 ± 3.49	21.85 ± 2.24
	G3 (TC + MT)	21.86 ± 4.57	20.98 ± 2.81	21.56 ± 2.46	21.98 ± 2.59	21.87 ± 2.87	22.54 ± 4.57	21.98 ± 3.96
	G4 (TC + AO)	20.54 ± 2.69	21.75 ± 3.51	21.78 ± 3.25	21.83 ± 3.24	21.95 ± 3.13	22.61 ± 2.78	22.23 ± 2.84
Fat (%)	G1 (control)	4.43 ± 0.38	4.32 ± 0.42	4.72 ± 0.58	4.25 ± 0.42	4.68 ± 0.34	4.74 ± 0.62	4.55 ± 0.49
	G2 (TC)	4.62 ± 0.40	4.48 ± 0.39	4.51 ± 0.47	4.41 ± 0.35	4.40 ± 0.45	4.47 ± 0.48	4.54 ± 0.23
	G3 (TC + MT)	4.54 ± 0.50	4.51 ± 0.42	4.53 ± 0.64	4.48 ± 0.47	4.47 ± 0.41	4.42 ± 0.31	4.48 ± 0.28
	G4 (TC + AO)	4.68 ± 0.54	4.62 ± 0.56	4.58 ± 0.43	4.51 ± 0.59	4.54 ± 0.37	4.51 ± 0.43	4.51 ± 0.34
Protein (%)	G1 (control)	3.07 ± 0.05	3.12 ± 0.06	3.10 ± 0.04	3.06 ± 0.07	3.11 ± 0.05	3.05 ± 0.06	3.08 ± 0.04
	G2 (TC)	3.17 ± 0.03	3.20 ± 0.14	3.21 ± 0.05	3.21 ± 0.08	3.25 ± 0.06	3.28 ± 0.04	3.24 ± 0.06
	G3 (TC + MT)	3.08 ± 0.06	3.15 ± 0.05	3.14 ± 0.04	3.18 ± 0.07	3.18 ± 0.04	3.16 ± 0.14	3.20 ± 0.07
	G4 (TC + AO)	3.24 ± 0.05	3.28 ± 0.06	3.25 ± 0.05	3.31 ± 0.05	3.30 ± 0.06	3.27 ± 0.04	3.30 ± 0.06
Lactose (%)	G1 (control)	4.37 ± 0.14	4.30 ± 0.15	4.36 ± 0.18	4.37 ± 0.12	4.42 ± 0.13	4.35 ± 0.17	4.45 ± 0.14
	G2 (TC)	4.41 ± 0.15	4.45 ± 0.14	4.48 ± 0.11	4.41 ± 0.11	4.46 ± 0.12	4.34 ± 0.13	4.37 ± 0.15
	G3 (TC + MT)	4.46 ± 0.13	4.44 ± 0.12	4.40 ± 0.15	4.39 ± 0.12	4.42 ± 0.17	4.45 ± 0.15	4.49 ± 0.14
	G4 (TC + AO)	4.54 ± 0.12	4.51 ± 0.13	4.57 ± 0.12	4.58 ± 0.13	4.51 ± 0.14	4.53 ± 0.14	4.52 ± 0.13
SNF (%)	G1 (control)	8.71 ± 0.13	8.67 ± 0.13	8.72 ± 0.17	8.87 ± 0.16	8.76 ± 0.19	8.68 ± 0.14	8.73 ± 0.18
	G2 (TC)	8.67 ± 0.21	8.63 ± 0.14	8.59 ± 0.13	8.62 ± 0.17	8.63 ± 0.13	8.65 ± 0.16	8.68 ± 0.13
	G3 (TC + MT)	8.85 ± 0.18	8.83 ± 0.16	8.79 ± 0.12	8.81 ± 0.15	8.73 ± 0.16	8.83 ± 0.19	8.81 ± 0.16
	G4 (TC + AO)	8.74 ± 0.15	8.70 ± 0.18	8.73 ± 0.15	8.69 ± 0.12	8.75 ± 0.17	8.75 ± 0.17	8.80 ± 0.15

Holstein dairy cows were assigned to 30 kg of TMR with no TC cows (G1 group; *n =* 8), 90 gm of TC (G2 group; *n =* 8), 54 gm of TC + 36 gm of MT (G3 group; *n =* 8) and 54 gm of TC + 36 gm of AO 40% (G4 group; *n =* 8), respectively.

## Discussion

Dandelion (Taraxacum) is recognized for its abundant mineral and vitamin content, particularly high levels of vitamins A and C, and iron [15]. Among *Taraxacum* species, *T. officinale, *the so-called “western dandelion,” is well known and widely used as a functional health food and its antioxidant efficacy has been well investigated [16]. There were no differences in the nutrient compositions, crude ash, protein, or carbohydrate contents of the aerial parts (flowers, leaves, and stems) of *T. coreanum *and* T. officinale*n [17]. However, the levels of vitamins A, E, and C are significantly higher in *T. coreanum* than *T. officinale* [17], and vitamins C and E are confirmed antioxidants [18]. Additionally, *T. coreanum *contains abundant flavonoids, which are representative phenolic compounds derived from plants and well-known antioxidants [5,6]. Oh et al. [8] have reported that *T. coreanum* contains more phenolic compounds and exhibits better antioxidant activity than *T. ofﬁcinale*. Therefore, *T. coreanum*, a Korean domestic dandelion, might be more advantageous in development as a feed additive than *T. ofﬁcinale*. Furthermore, aerial parts of *T. coreanum* can be harvested approximately 5 times a year when the roots are left in the ground because the aerial parts regrow from the remaining roots. Moreover, the total polyphenol and flavonoid contents and antioxidative activity of *T. coreanum* extracts are higher in the aerial parts than in the roots [19]. In addition, the naturally dried form has a lower production cost than the extract form and can act as a coarse fiber for dairy cows. Thus, natural drying of the aerial part of *T. coreanum* could be an optimal choice in terms of economy and efficacy when used as an additive feed for the management of lactating dairy cows.

**Table 3.** Changes in blood corpuscle compositions in Holstein cows during the 6-week administration period.

Parameters	Group	0w	1w	2w	3w	4w	5w	6w
WBC (×10^9^/l)	G1 (control) (Control(control)	22.4 ± 2.7	23.9 ± 1.1	19.6 ± 4.8	23.0 ± 1.2	19.9 ± 1.7	19.0 ± 5.2	21.1 ± 2.7
	G2 (TC)	21.2 ± 3.3	21.4 ± 1.5	20.0 ± 3.5	17.6 ± 4.8	15.8 ± 3.2	14.3 ± 4.0	14.4 ± 2.2*
	G3 (TC + MT)	20.7 ± 1.3	21.3 ± 1.5	17.5 ± 2.6	17.9 ± 8.7	15.4 ± 3.0	15.3 ± 8.1	13.2 ± 3.0*
	G4 (TC + AO)	22.3 ± 4.8	22.3 ± 3.5	20.4 ± 4.2	19.3 ± 2.3	18.7 ± 4.2	16.4 ± 4.1	12.5 ± 3.1*
RBC (×10^12^/l)	G1 (control)	6.7 ± 0.3	6.9 ± 0.2	6.8 ± 0.7	7.0 ± 1.0	7.0 ± 0.7	7.7 ± 1.1	7.3 ± 0.8
	G2 (TC)	6.4 ± 1.0	6.6 ± 1.1	6.9 ± 0.8	6.8 ± 1.5	7.8 ± 2.6	7.0 ± 0.8	6.9 ± 1.0
	G3 (TC + MT)	5.7 ± 0.2	5.6 ± 0.1	7.2 ± 0.7	8.6 ± 3.0	7.0 ± 0.5	6.1 ± 1.1	6.9 ± 1.7
	G4 (TC + AO)	5.3 ± 0.8	6.5 ± 0.9	6.6 ± 0.5	8.5 ± 1.3	8.1 ± 3.1	6.8 ± 1.1	6.7 ± 1.0
HGB (gm/dl)	G1 (control)	9.2 ± 0.5	10.3 ± 0.4	10.7 ± 0.9	14.0 ± 0.6	14.0 ± 1.1	14.5 ± 0.9	13.6 ± 0.6
	G2 (TC alone)	9.9 ± 0.7	10.3 ± 0.8	11.1 ± 4.7	11.7 ± 2.4	14.1 ± 1.2	12.6 ± 0.9	13.0 ± 0.4
	G3 (TC + MT)	8.9 ± 0.7	8.6 ± 0.7	11.8 ± 1.7	15.7 ± 6.2	12.4 ± 0.8	12.7 ± 1.1	12.7 ± 1.4
	G4 (TC + AO)	8.6 ± 0.7	9.0 ± 0.6	10.5 ± 0.6	14.9 ± 2.4	13.6 ± 1.6	12.5 ± 2.2	11.6 ± 2.1
HCT (%)	G1 (control)	36.7 ± 2.3	37.8 ± 2.0	37.6 ± 2.4	41.5 ± 3.9	41.5 ± 3.4	44.6 ± 2.9	44.1 ± 3.8
	G2 (TC)	36.0 ± 3.1	37.4 ± 3.1	40.6 ± 1.7	40.5 ± 2.9	44.0 ± 2.2	40.5 ± 3.4	37.9 ± 7.6
	G3 (TC + MT)	34.2 ± 2.0	33.3 ± 1.5	43.1 ± 2.0	41.5 ± 3.3	41.7 ± 0.9	31.1 ± 8.5	34.3 ± 7.8
	G4 (TC + AO)	32.1 ± 3.2	33.2 ± 1.2	39.0 ± 1.3	41.8 ± 2.4	40.8 ± 1.0	40.2 ± 7.4	39.8 ± 6.4
MCV (fl)	G1 (control)	53.1 ± 1.4	60.9 ± 1.1	61.3 ± 2.5	59.8 ± 5.0	59.8 ± 4.9	58.2 ± 5.2	60.7 ± 2.6
	G2 (TC)	56.5 ± 4.3	57.2 ± 4.9	59.7 ± 4.5	59.9 ± 4.4	58.9 ± 4.9	57.2 ± 5.5	57.2 ± 3.7
	G3 (TC + MT)	59.8 ± 1.9	59.0 ± 1.8	60.2 ± 4.3	59.9 ± 3.9	59.5 ± 3.8	60.3 ± 3.0	61.4 ± 7.8
	G4 (TC + AO)	51.6 ± 5.5	51.6 ± 5.5	59.0 ± 2.7	60.8 ± 2.7	59.2 ± 2.1	58.8 ± 1.5	59.3 ± 1.3
MCH (Pg)	G1 (control)	17.8 ± 0.8	18.1 ± 0.0	19.1 ± 0.7	20.2 ± 2.7	20.2 ± 1.7	18.2 ± 2.1	18.8 ± 1.4
	G2 (TC)	15.4 ± 1.6	15.6 ± 1.4	15.9 ± 6.4	17.3 ± 1.4	18.0 ± 1.8	17.5 ± 1.5	14.5 ± 5.0
	G3 (TC + MT)	15.5 ± 1.1	15.2 ± 1.4	16.3 ± 1.3	18.1 ± 1.7	17.7 ± 1.4	17.6 ± 3.6	16.2 ± 1.1
	G4 (TC + AO)	13.8 ± 1.0	13.8 ± 1.0	15.9 ± 1.4	17.5 ± 1.0	18.2 ± 0.9	18.2 ± 0.4	17.2 ± 0.5
MCHC (gm/dl)	G1 (control)	31.5 ± 1.2	29.8 ± 0.5	30.9 ± 0.3	31.8 ± 2.8	32.8 ± 0.3	32.5 ± 0.6	31.0 ± 1.2
	G2 (TC)	27.3 ± 0.9	27.4 ± 0.2	27.0 ± 1.2	29.0 ± 1.2	30.5 ± 0.7	30.7 ± 0.4	28.4 ± 1.0
	G3 (TC + MT)	28.0 ± 1.3	26.9 ± 0.8	27.2 ± 0.3	30.2 ± 1.2	29.7 ± 0.9	29.2 ± 0.8	28.1 ± 0.4
	G4 (TC + AO)	27.0 ± 0.9	28.0 ± 1.1	28.0 ± 1.2	28.8 ± 1.0	30.8 ± 1.5	31.1 ± 0.3	29.0 ± 0.6
PLT (×10^9^/l)	G1 (control)	479.3 ± 149.7	619.0 ± 186.1	599.0 ± 106.4	510.0 ± 50.5	488.5 ± 150.0	552.7 ± 172.7	559.0 ± 135.3
	G2 (TC)	460.0 ± 122.0	582.5 ± 120.5	512.8 ± 118.5	490.3 ± 72.4	489.3 ± 141.3	530.0 ± 139.6	599.5 ± 162.1
	G3 (TC + MT)	484.5 ± 152.4	581.0 ± 147.5	562.0 ± 91.5	483.0 ± 86.5	461.0 ± 160.0	572.5 ± 175.7	571.7 ± 161.4
	G4 (TC + AO)	516.0 ± 129.9	584.5 ± 159.1	532.7 ± 70.1	524.8 ± 87.5	454.8 ± 100.4	518.4 ± 196.7	548.7 ± 108.7

Holstein dairy cows were assigned to 30 kg of TMR with no TC cows (G1 group; *n =* 8), 90 gm of TC (G2 group; *n =* 8), 54 gm of TC + 36 gm of MT (G3 group; *n =* 8) and 54 gm of TC + 36 gm of AO 40% (G4 group; *n =* 8), respectively.

* Significant difference compared to the control group (*p* < 0.05).

In the dairy cow industry, commercial lactation diets are generally high in concentrate to maximize nutrient intake and milk production, resulting in metabolic and systemic dysfunction and an increased occurrence of subacute ruminal acidosis [20]. Oxidative stress, indicative of an imbalance between oxidant and antioxidant status, emerges during early lactation in cows, heightening the risk of various diseases by disrupting inflammatory responses [21]. Lactating cows are particularly susceptible to exacerbated oxidative stress [22], marked by an imbalance between the production of reactive oxygen species (ROS) and their elimination by protective mechanisms; prolonged oxidative stress can lead to chronic inflammation [23]. Among the antioxidant biomarkers, GSH-Px and SOD play pivotal roles in defending cells against elevated ROS levels [24]. Previously, Holbrook and Hicks [25] observed no significant fluctuation in SOD concentration throughout the lactation of non-mastitic cows, and Pilarczyk et al. [26] found the highest GSH-Px level in the early lactation stage, which may be in accordance with a rapid dietary transition to a high-energy feed postpartum. Therefore, significantly higher antioxidant biomarker (SOD and GSH-px) levels in TC-treated cows (G2, G3, and G4 groups) suggested a higher capacity to deal with oxidative stress during the later administration period, and a higher oxidative stress status could be eliminated because the experimental cows were fed identical TMR feed during the administration period in this study.

**Table 4.** Changes in serum chemistry parameters in Holstein cows during the 6-week administration period.

Parameters	Group	0w	1w	2w	3w	4w	5w	6w
AST (IU/l)	G1 (control)	38.51 ± 7.6	42.4 ± 6.9	45.7 ± 9.4	40.4 ± 8.4	47.8 ± 5.9	39.5 ± 11.6	41.3 ± 9.4
	G2 (TC alone)	37.2 ± 8.4	37.7 ± 7.5	39.4 ± 11.8	38.6 ± 10.6	37.5 ± 9.2	39.9 ± 10.4	40.2 ± 5.7
	G3 (TC + MT)	31.9 ± 6.6	34.5 ± 4.9	30.8 ± 7.1	47.5 ± 7.2	40.4 ± 7.6	42.2 ± 8.5	41.5 ± 6.4
	G4 (TC + AO)	42.3 ± 9.8	47.5 ± 7.2	45.8 ± 6.8	43.1 ± 5.9	44.6 ± 8.1	41.6 ± 5.9	42.8 ± 4.9
ALT (IU/l)	G1 (control)	55.8 ± 14.4	53.6 ± 9.2	48.8 ± 7.6	53.4 ± 7.6	43.8 ± 11.3	42.6 ± 8.0	44.2 ± 13.1
	G2 (TC alone)	42.8 ± 13.5	47.1 ± 10.9	47.4 ± 13.7	47.6 ± 9.3	47.7 ± 12.6	45.8 ± 9.4	44.4 ± 9.91
	G3 (TC + MT)	55.8 ± 14.4	53.4 ± 7.6	48.8 ± 7.6	55.8 ± 14.4	43.5 ± 9.8	37.4 ± 9.9	36.5 ± 9.1
	G4 (TC + AO)	50.0 ± 7.5	54.6 ± 5.0	49.6 ± 2.7	48.8 ± 9.4	43.0 ± 10.2	42.8 ± 6.7	38.4 ± 12.3
ALP (IU/l)	G1 (control)	296.0 ± 59.6	220.2 ± 25.9	202.0 ± 13.1	160.4 ± 27.7	152.40 ± 15.3	145.80 ± 21.6	123.0 ± 11.7
	G2 (TC alone)	275.8 ± 41.8	231.2 ± 30.5	253.2 ± 66.1	190.0 ± 22.3	148.40 ± 34.1	133.60 ± 32.7	120.6 ± 15.1
	G3 (TC + MT)	214.7 ± 32.1	269.0 ± 23.0	266.0 ± 36.9	181.8 ± 38.3	178.20 ± 27.0	168.60 ± 12.7	144.0 ± 35.7
	G4 (TC + AO)	227.5 ± 44.9	245.5 ± 17.6	284.5 ± 21.9	164.8 ± 27.1	184.40 ± 18.5	128.20 ± 13.4	145.4 ± 23.3
TP (gm/dl)	G1 (control)	8.6 ± 0.5	8.8 ± 0.4	9.1 ± 0.6	8.9 ± 0.4	8.7 ± 0.5	8.6 ± 0.3	8.9 ± 0.5
	G2 (TC alone)	8.0 ± 0.4	8.4 ± 0.3	8.3 ± 0.3	8.5 ± 0.6	8.6 ± 0.3	8.9 ± 0.4	8.7 ± 0.3
	G3 (TC + MT)	8.3 ± 0.4	8.5 ± 0.6	8.7 ± 0.5	8.5 ± 0.4	8.9 ± 0.3	8.6 ± 0.4	8.7 ± 0.3
	G4 (TC + AO)	8.7 ± 0.6	8.6 ± 0.4	8.9 ± 0.7	9.0 ± 0.3	8.7 ± 0.4	8.7 ± 0.3	8.8 ± 0.4
ALB (gm/dl)	G1 (control)	3.7 ± 0.3	3.8 ± 0.2	3.6 ± 0.2	3.8 ± 0.4	3.9 ± 0.3	4.0 ± 0.4	3.8 ± 0.3
	G2 (TC alone)	3.6 ± 0.3	3.8 ± 0.4	3.9 ± 0.5	4.0 ± 0.4	3.8 ± 0.4	4.0 ± 0.2	3.9 ± 0.4
	G3 (TC + MT)	3.6 ± 0.2	3.7 ± 0.1	3.9 ± 0.5	3.9 ± 0.2	3.7 ± 0.3	3.9 ± 0.6	3.7 ± 0.4
	G4 (TC + AO)	3.5 ± 0.4	3.5 ± 0.2	4.1 ± 0.4	3.8 ± 0.4	4.0 ± 0.2	3.8 ± 0.3	4.0 ± 0.3
GLU (mg/dl)	G1 (control)	100.8 ± 3.6	110.8 ± 15.8	100.6 ± 3.2	108.00 ± 9.0	111.2 ± 8.7	103.4 ± 10.9	104.0 ± 9.5
	G2 (TC alone)	121.8 ± 9.6	118.0 ± 5.6	103.0 ± 6.5	106.40 ± 4.9	99.0 ± 9.0	97.4 ± 7.8	90.6 ± 6.3
	G3 (TC + MT)	121.2 ± 15.8	112.8 ± 9.3	103.6 ± 9.3	110.20 ± 6.8	103.0 ± 17.0	95.4 ± 7.7	98.8 ± 7.5
	G4 (TC + AO)	102.0 ± 12.1	108.0 ± 14.2	99.0 ± 7.2	101.0 ± 10.0	96.8 ± 12.4	98.00 ± 6.6	90.4 ± 3.3
LDH (IU/l)	G1 (control)	863.6 ± 86.4	727.5 ± 140.1	852.4 ± 99.7	611.2 ± 54.2	447.2 ± 41.4	557.6 ± 108.2	505.6 ± 72.9
	G2 (TC alone)	988.7 ± 53.1	741.0 ± 120.7	889.0 ± 70.4	596.7 ± 38.8	504.0 ± 42.2	568.6 ± 154.7	495.0 ± 40.7
	G3 (TC + MT)	942.0 ± 63.6	737.6 ± 136.2	754.7 ± 92.8	648.4 ± 41.7	462.0 ± 64.8	568.2 ± 110.2	469.5 ± 47.8
	G4 (TC + AO)	901.5 ± 74.0	707.5 ± 132.1	818.5 ± 105.2	548.4 ± 129.9	515.5 ± 37.0	527.0 ± 13.4	506.5 ± 36.0
T-BIL (mg/dl)	G1 (control)	0.03 ± 0.01	0.04 ± 0.01	0.07 ± 0.02	0.05 ± 0.03	0.06 ± 0.02	0.05 ± 0.01	0.04 ± 0.00
	G2 (TC alone)	0.03 ± 0.02	0.02 ± 0.00	0.02 ± 0.01	0.04 ± 0.02	0.03 ± 0.03	0.03 ± 0.03	0.03 ± 0.01
	G3 (TC + MT)	0.03 ± 0.02	0.03 ± 0.01	0.04 ± 0.02	0.03 ± 0.01	0.04 ± 0.02	0.04 ± 0.01	0.05 ± 0.02
	G4 (TC + AO)	0.04 ± 0.01	0.03 ± 0.01	0.05 ± 0.01	0.04 ± 0.01	0.05 ± 0.02	0.03 ± 0.01	0.04 ± 0.00
T-CHO (mg/dl)	G1 (control)	103.4 ± 13.5	91.2 ± 13.8	79.4 ± 9.63	79.4 ± 5.2	91.8 ± 12.5	109.4 ± 9.7	108.8 ± 11.5
	G2 (TC alone)	104.8 ± 15.2	109.6 ± 9.5	88.8 ± 10.71	82.2 ± 5.8	96.6 ± 3.5	104.0 ± 10.2	105.4 ± 10.0
	G3 (TC + MT)	97.6 ± 7.6	99.4 ± 13.9	78.6 ± 4.51	85.8 ± 6.3	88.2 ± 10.8	101.6 ± 9.2	109.4 ± 9.5
	G4 (TC + AO)	107.2 ± 10.7	102.5 ± 13.3	86.5 ± 5.8	90.4 ± 7.9	94.5 ± 7.6	108.8 ± 7.8	105.2 ± 9.8
TG (mg/dl)	G1 (control)	59.33 ± 17.24	57.80 ± 21.09	54.40 ± 17.70	40.60 ± 10.38	55.60 ± 17.10	48.00 ± 9.6	52.0 ± 6.9
	G2 (TC alone)	56.00 ± 7.52	51.20 ± 10.76	63.40 ± 13.81	47.80 ± 8.44	44.00 ± 20.21	45.40 ± 18.2	49.6 ± 13.4
	G3 (TC + MT)	57.40 ± 11.52	57.40 ± 11.52	42.40 ± 17.44	46.60 ± 13.59	41.50 ± 6.66	46.60 ± 12.7	52.6 ± 17.3
	G4 (TC + AO)	51.80 ± 16.42	49.60 ± 6.58	50.20 ± 11.12	41.80 ± 10.42	38.00 ± 4.24	48.5 ± 9.1	50.5 ± 17.6
BUN (mg/dl)	G1 (control)	9.2 ± 0.7	9.70 ± 2.0	9.7 ± 1.0	11.8 ± 1.1	11.2 ± 0.7	11.3 ± 1.3	10.8 ± 1.1
	G2 (TC alone)	8.1 ± 1.3	10.9 ± 2.4	10.1 ± 1.5	10.4 ± 3.2	10.1 ± 1.3	9.8 ± 1.1	10.5 ± 0.9
	G3 (TC + MT)	9.7 ± 2.8	11.7 ± 3.0	10.5 ± 1.2	10.1 ± 1.6	9.0 ± 1.9	10.5 ± 1.2	9.8 ± 1.0
	G4 (TC + AO)	9.2 ± 1.0	11.1 ± 1.8	11.9 ± 1.6	11.2 ± 1.9	10.4 ± 1.3	11.3 ± 1.3	10.6 ± 0.9
CRE (mg/dl)	G1 (control)	0.69 ± 0.12	0.75 ± 0.26	0.81 ± 0.10	0.86 ± 0.07	1.02 ± 0.45	1.07 ± 0.12	1.12 ± 0.16
	G2 (TC alone)	0.78 ± 0.28	0.84 ± 0.26	0.80 ± 0.15	0.87 ± 0.21	0.89 ± 0.16	1.24 ± 0.24	1.08 ± 0.13
	G3 (TC + MT)	0.71 ± 0.20	0.75 ± 0.15	0.79 ± 0.20	0.83 ± 0.11	0.99 ± 0.43	1.18 ± 0.28	1.13 ± 0.18
	G4 (TC + AO)	0.78 ± 0.15	0.82 ± 0.11	0.81 ± 0.21	0.85 ± 0.12	1.01 ± 0.34	1.15 ± 0.23	1.10 ± 0.17

Holstein dairy cows were assigned to 30 kg of TMR with no TC cows (G1 group; *n =* 8), 90 gm of TC (G2 group; *n =* 8), 54 gm of TC + 36 gm of MT (G3 group; *n =* 8) and 54 gm of TC + 36 gm of AO 40% (G4 group; *n =* 8), respectively.

The concentrations of pro-inflammatory cytokines (TNF-α) and chemokines (IL-8) were significantly lower in TC-treated cows during the latter administration period, corresponding with the increased antioxidant levels. TNF-α, a primary pro-inflammatory cytokine, orchestrates immune responses by stimulating the proliferation, differentiation, and activity of numerous immune cells [27], while IL-8, also known as neutrophil-activating peptide 1, facilitates neutrophil migration to sites of inflammation, promoting increased phagocytosis during the inflammatory phase [28]. As a result, these elements encourage the diapedesis of neutrophils and monocytes, along with chemoattraction, thus fostering heightened phagocytic activity during the inflammatory phase [29]. In the present study, no clinical symptoms such as mastitis, diarrhea, or pneumonia were observed in any experimental animals before, during, or after the experimental period. Therefore, we concluded that the reduced inflammatory response was due to increased oxidative activity caused by *T. coreanum* feeding.

The final goal of dairy farm management is to improve milk yield and quality. Among the factors affecting milk quality, SCC is key in determining milk grade, and a significant increase in SCC (5%–20%) is associated with abnormalities in udder health, decreased milk quality, and milk production loss [30]. Therefore, decreasing the SCC in milk composition suggests an improvement in milk yield, quality, and udder health. In the present study, the three types of feed additives composed of *T. coreanum *(TC, mixtures of TC + MT, or TC + AO) showed significantly lower SCC levels during the latter administration period, along with a significantly higher antioxidative capacity during a similar period. Therefore, dried *T. coreanum* and its mixture with other medicinal herbs are suitable for development as a feed additive for lactating Holstein dairy cows. It is a promising substance for improving milk quality, and feeding for 4 weeks is highly recommended.

## Conclusion

Supplementation of dried *T. coreanum *and *T. coreanum *mixtures with MT or AO for 4 weeks enhanced the antioxidative activities in the blood due to significantly higher antioxidative biomarkers (SOD and GSH-px). In addition, they elicited the lower serum proinflammatory cytokines (TNF-α) and chemokine (IL-8) release supported by lower WBC and SCC counts during the administration period. Therefore, they exhibited useful efficacy as feed additives by enhancing the serum antioxidative capacity in accordance with the reduced inflammatory response.

## List of Abbreviations

ALB, albumin; ALP, alkaline phosphatase; ALT, alanine aminotransaminase; AO, *Aspergillus oryzae*; AST, aspartate transaminase; BUN, blood urea nitrogen; CBC, complete blood cell count; CRE, creatinine; GLU, glucose; HCT, hematocrit; HGB, hemoglobin; HSD, honest significant difference; IL-8, interleukin-8; LDH, lactate dehydrogenase; MCH, mean corpuscular hemoglobin; MCHC, mean corpuscular hemoglobin concentration; MCV, mean corpuscular volume; MT, Milk thistle; PLT, platelet; RBC, red blood cells; ROS, reactive oxygen species; SCC, somatic cell count; SNF, solids not fat; SOD, superoxide dismutase; T-BIL, total bilirubin; TC, *Taraxacum coreanum*; T-CHOL, total cholesterol; TG, triglyceride; TMR, total mixed ration; TNF- α, tumor necrosis factor alpha; TP, total protein; WBC, white blood cells.
